# Anterosuperior approach versus deltopectoral approach for reverse total shoulder arthroplasty: a systematic review and meta-analysis

**DOI:** 10.1186/s13018-022-03414-9

**Published:** 2022-12-08

**Authors:** Hyun-Gyu Seok, Jeong Jin Park, Sam-Guk Park

**Affiliations:** Department of Orthopedic Surgery, Yeungnam University Hospital, Yeungnam University Medical Center, 170 Hyeonchung-Ro, Nam-Gu, Daegu, 42415 Republic of Korea

**Keywords:** Deltopectoral approach, Anterosuperior approach, Reverse total shoulder arthroplasty, Meta-analysis

## Abstract

**Background:**

Surgical approach is an important factor that may affect the outcomes of reverse total shoulder arthroplasty (RTSA). The most common approaches for RTSA are anterosuperior (AS) and deltopectoral (DP). However, controversy exists on which surgical approach is better. This meta-analysis aimed to compare both approaches in terms of radiological and clinical outcomes and complications.

**Methods:**

We searched PubMed, Embase, and Cochrane Library databases for studies that compared the postoperative outcomes of the AS and DP approaches for RTSA. After screening and quality assessment of the articles, we obtained two randomized controlled trials and four retrospective comparative studies. We analyzed the radiologic outcomes, functional outcomes, and complications between the two approaches. The standardized mean difference and odds ratio were used to analyze the differences in outcomes between the two surgical approaches. Statistical significance was set at *P* < 0.05.

**Results:**

The incidence rate of glenoid implant loosening was significantly (*P* = 0.04) lower in the AS group than that in the DP group. In terms of forward flexion after surgery, the DP approach produced significantly (*P* = 0.03) better outcomes compared with the AS approach. There were no significant differences in radiological outcomes or other complication rates between the two approaches.

**Conclusion:**

As a result of this meta-analysis, one of the two approaches did not bring a better result than the other. One has strength for better forward flexion and the other for a lower glenoid loosening rate. With this in mind, it is recommended to use the approach that the surgeon is most familiar with.

## Background

Reverse total shoulder arthroplasty (RTSA) is a common surgical option for cuff tear arthropathy and osteoarthritis (OA) with rotator cuff tears [1–3]. With advanced understanding of biomechanics and the development of prostheses, the use of RTSA has recently been extended to acute fractures, primary OA, arthroplasty revision surgery, and tumoral surgery [4, 5]. The two most commonly used approaches for RTSA are anterosuperior (AS) and deltopectoral (DP) [3, 6, 7]. Both approaches are associated with few advantages and disadvantages. The AS approach preserves the subscapularis tendon and straight exposure of the glenoid but the device positioning is difficult [6, 8–10]. The DP approach preserves the deltoid and pectoralis origins; however, the approach requires resection of the subscapularis [6, 10]. Although several studies have attempted to determine the optimal surgical approach for RTSA [3, 6, 11], results were inconsistent, precluding a conclusion on the better approach. In addition, comparison has been made between the two approaches, but no meta-analysis has analyzed the radiological and clinical outcomes and complications [12]. Hence, we aimed to compare the two surgical approaches, AS and DP, for RTSA using a meta-analysis including suitable comparative studies.

## Materials and methods

This meta-analysis was conducted in accordance with the Preferred Reporting Items for Systematic Reviews and Meta-Analysis (PRISMA) guidelines [13].

### Literature search strategy and study selection

Relevant studies published between January 1, 2000, and September 30, 2022, were systematically searched for in PubMed, Embase, and the Cochrane Library. The search included a combination of different terms and synonyms: ('deltopectoral approach') AND 'anterosuperior approach' or ‘superior approach’ or ‘superolateral approach’) AND ('reverse total shoulder arthroplasty' or 'reverse total shoulder replacement'). The DP approach is used as a unified word, but the AS approach is also used as a superior or superolateral approach. So, for each article, the authors decided whether to include the approach after checking the description in the text. In addition, reference lists of previously published review articles were manually searched for additional eligible studies.

We applied the following inclusion criteria for the selection of articles: (1) studies describing the clinical or radiological outcomes of the AS and DP approaches for RTSA; (2) quantitative studies, such as comparative and randomized controlled studies; (3) studies with adequate data for analysis; and (4) studies with a follow-up period of at least 12 months.

The exclusion criteria were as follows: (1) case reports, reviews, or other indistinct forms; (2) studies that repeatedly published the same data; (3) follow-up of less than 12 months; and (4) studies with no reports on study outcomes.

### Data extraction

**Fig. 1:**
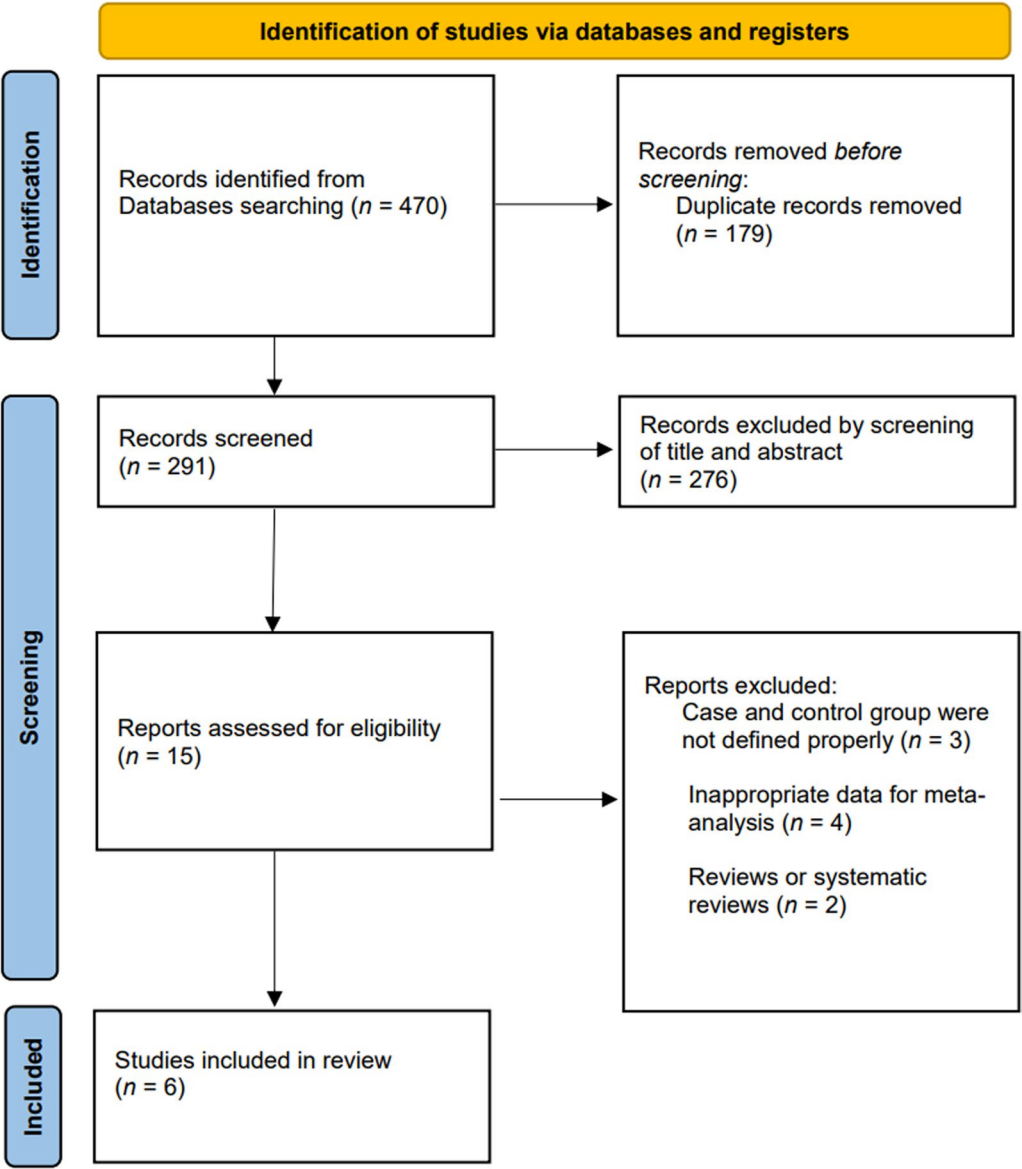
Flow chart for literature identification using the preferred reporting items for systematic reviews and meta-analyses (PRISMA) guidelines

We used the PRISMA flow chart (Fig. 1) to select the included studies; the results of literature search were imported into the Endnote X9. After discarding duplicate studies, two authors (H.G.S. and J.J.P.) independently assessed the potentially eligible studies. The remaining articles were screened for eligibility based on a review of their titles and abstracts. After screening, the full texts of the remaining potential studies were independently read by the two authors (H.G.S. and J.J.P.), and the eligibility of each article was reassessed. Disagreements were resolved through consensus. The conflicts were resolved by including a third author (S.G.P.). Subsequently, data including the first author, publication year, design of the study, demographic factors, number of patients, name of prosthesis used, follow-up duration, complications, and outcomes (radiologic and clinical) were extracted.

### Quality assessment

We included randomized controlled trials and retrospective comparative studies. The methodological quality of the included comparative studies was assessed using the Newcastle–Ottawa Scale (NOS) [14]. The quality of each study was graded as good, fair, or poor. All studies evaluated by the NOS were confirmed to be of good quality (Table 1). The risk of bias in the included randomized controlled trials was assessed using the risk of bias tool recommended by the Cochrane Collaboration [15] (Table 2).

**Table 1:** Quality assessment of the studies included in the meta-analysis based on the Newcastle–Ottawa scale

Authors	Selection				Comparability	Outcome			Total score
	Exposed cohort	non-exposed cohort	Ascertainment of exposure	outcome of interest		assessment of outcome	Length of follow-up	Adequacy of follow-up	
Aibinder et al. [3]	★	★	★	★	★★	★	★		8(good)
Choi et al. [11]	★	★	★	★	★★	★	★	★	9(good)
Gillespie et al. [9]	★	★	★	★	★	★	★	★	8(good)
Ladermann et al. [16]	★	★	★	★	★★	★	★		8(good)

**Table 2:** Quality assessment of the randomized controlled trials included in the meta-analysis based on the risk of bias tool recommended by the Cochrane Collaboration

Study	Random sequence generation	Allocation concealment	Blinding of participants and personnel	Blinding of outcome assessment	Incomplete outcome data	Selective reporting	Other bias
Ottini et al. [17]	Low risk	Low risk	Low risk	Low risk	Low risk	Low risk	Low risk
Torrens et al. [6]	Low risk	Low risk	Low risk	Low risk	Low risk	Low risk	Low risk

### Statistical analyses

The pooled data were collected and recorded into the RevMan 5.4 software for meta-analysis. A heterogeneity test was conducted during each analysis using *I*^2^ statistics to measure the extent of inconsistency among the results. A fixed-effects model was applied when homogeneity (*I*^2^ < 50%) was observed. In contrast, when the *I*^2^ value was ≥ 50%, a random effects model was applied.

The standard mean difference (SMD) was calculated for continuous outcomes, such as prosthesis-scapular neck angle (PSNA), glenosphere overhang, constant score, and degree of forward flexion. Odds ratios (OR) were calculated for dichotomous outcomes, such as complications. In addition, 95% confidence intervals (CIs) were used for analysis. Statistical significance was set at *p* < 0.05.

## Results

### Study selection and characteristics

A total of 470 potential studies were identified through PubMed (*n* = 164), Embase (*n* = 232), and the Cochrane Library (*n* = 74). After removing duplicates studies, the titles and abstracts of the remaining articles were initially reviewed, and 15 articles were considered appropriate for the next stage of review. Of the 15 articles that were possibly eligible for inclusion, 9 were excluded for reasons of “the papers were review articles” and some other reasons (details are shown in Fig. 1). Finally, six studies (four retrospective comparative studies and two randomized controlled studies) were included in the meta-analysis. One randomized controlled trial [6] provided continuous data, such as constant score, range of motion, and radiologic outcomes according to surgical indication (acute fracture and cuff rupture arthropathy). Although the data were of one study, it was set to a different group in the analysis using continuous data. The selected six studies included 241 and 225 cases in the AS and DP groups, respectively. Table 3 presents the detailed characteristics of each study.

**Table 3:** Characteristics of the included studies

Authors (Year)	Study design (LOE)	Mean age, years	Mean FU	AS, *n*	DP, *n*	Total, *n*	Prosthesis	Surgical indication	Outcomes recorded
Aibinder et al. [3] (2018)	Retrospective comparative (IV)	73.0	AS: 3.7 yrs DP: 2.4 yrs	87	22	109	NR	Cuff tear arthropathy, osteoarthritis with a rotator cuff tear	SNA, PSNA, PGRD, α, β tilt, scapular notching, complications, pain, ROM
Choi et al. [11] (2017)	Retrospective comparative (IV)	AS: 73.2 ± 7.2 DP: 70.8 ± 9.2	AS: 13.0 mo DP: 13.4 mo	12	12	24	SMR Reverse Shoulder Prosthesis (Lima)	Cuff tear arthropathy	PSNA, PGRD, overhang, scapular notching, VAS, ASES, constant score, ROM, complications
Gillespie et al. [9] (2015)	Retrospective comparative (IV)	NR	3.1 yrs	45	19	64	Grammont-style prosthesis	Cuff tear arthropathy, osteoarthritis with a rotator cuff tear, failed rotator cuff repair, rheumatoid arthritis, proximal humerus malunion	ROM, SANE, PSS, SST, position of prosthesis, radiographic notching, complications
Ladermann et al. [16] (2011)	Retrospective comparative (IV)	AS: 75.1 ± 6.4 DP: 72.8 ± 8.9	AS: 19.7 ± 12.9 mo DP: 18.3 ± 14.0 mo	35	109	144	Reversed Aequalis implant (Tornier)	Cuff tear arthropathy or osteoarthritis with a rotator cuff tear, rheumatoid arthritis, trauma, avascular necrosis, recurrent glenohumeral instability	forward flexion, arm lengthening compared to contralateral side
Ottini et al. [17] (2017)	Randomized controlled trial (II)	75	At least 2 years	13	14	27	Anatomical Reverse® prosthesis (Zimmer)	Cuff tear arthropathy, osteoarthritis with a rotator cuff tear, massive rotator cuff tear	Constant score, patient’s stasifatcion, ROM, glenoid inclination, scapular notching, complications
Torrens et al. [6] (2021)	Randomized controlled trial (II)	74.4 ± 6.3	At least 2 years	49	49	98	Delta Xtend RSA (DePuy)	Cuff tear arthropathy, acute fracture	glenosphere overhang, glenosphere tilt, PSNA, Constant score, scapular notching, forward flexion, complications

### Radiologic outcomes

**Fig. 2:**
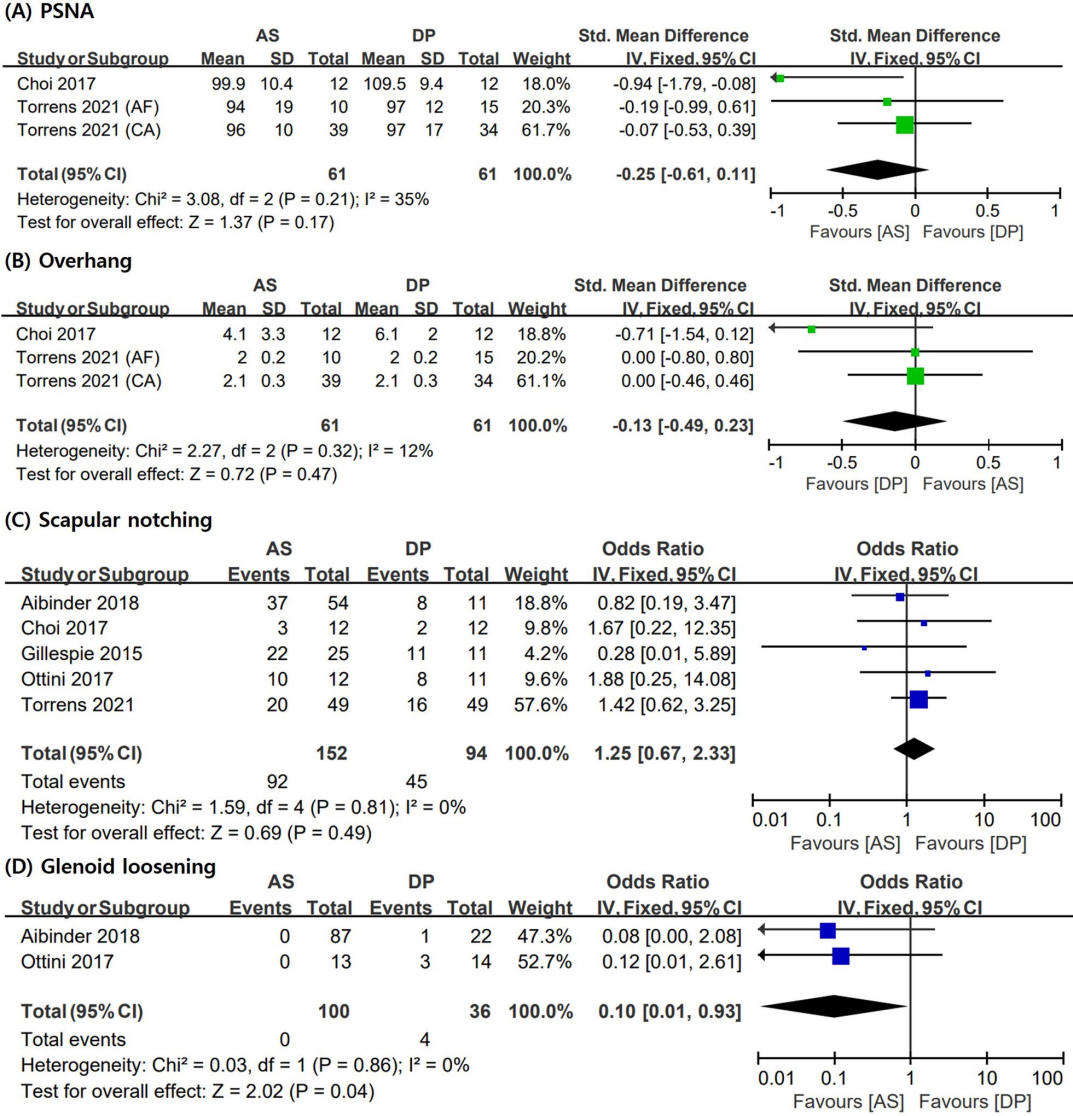
Meta-analysis results in terms of radiologic outcomes: **A** PSNA, **B** overhang, **C** scapular notching, and **D** glenoid loosening. AS: anterosuperior approach; DP: deltopectoral approach; AF: acute fracture; CA: cuff tear arthropathy; SD: standard deviation; PSNA: prosthesis-scapular neck angle

The measured values of radiological outcomes, such as PSNA and overhang, were evaluated in three of the studies included in this meta-analysis. Among these, only two studies [6, 11] provided suitable data for analysis. In one of these two studies, Torrens et al. [6] presented values according to surgical indication (acute fracture and cuff tear arthropathy). No statistically significant difference was observed in PSNA (SMD = − 0.25; 95% CI = − 0.61–0.11; I2 = 35%) and overhang (SMD = − 0.13; 95% CI = − 0.49–0.23; I2 = 12%) between the AS and DP groups, and the fixed-effect model was used for the analyses. An analysis of scapular notching, the most common radiological adverse event of RTSA, was also performed. Data on scapular notching were described in all studies included in the analysis except for one study [3, 6, 9, 11, 17]. No statistically significant difference was observed in scapular notching (pooled OR = 1.25; 95% CI = 0.67–2.33; I2 = 0%) between the two groups. In contrast, the AS group had a lower incidence of glenoid implant loosening (pooled OR = 0.10; 95% CI = 0.01–0.93; I2 = 0%) than that of the DP group. Figure 2 shows the forest plots, SMD, OR, 95% CI, and heterogeneity for radiological outcomes.

### Clinical outcomes

**Fig. 3:**
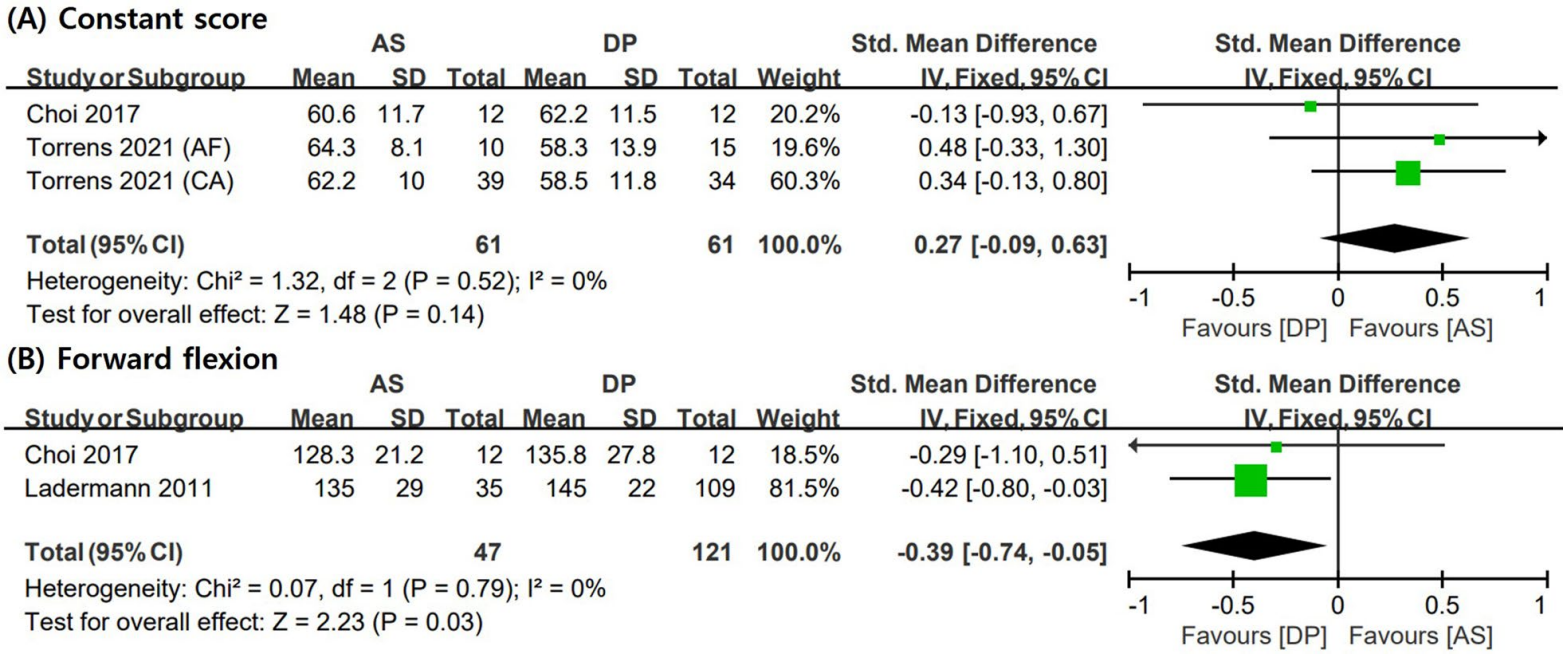
Meta-analysis results in terms of clinical outcomes: **A** constant score and **B** forward flexion. AS: anterosuperior approach; DP: deltopectoral approach; AF: acute fracture; CA: cuff tear arthropathy; SD: standard deviation

Figure 3 shows the forest plots, SMD, 95% CI, and heterogeneity for clinical outcomes such as constant score and forward flexion. Two studies [6, 11] compared AS and DP using constant scores, while two [11, 16] compared the groups using forward flexion of the shoulder. The fixed-effect model was used for the analysis of clinical outcomes. The measured forward flexion of the shoulder (SMD = − 0.39; 95% CI = − 0.74, − 0.05; I2 = 0%) was significantly higher in the DP group than that in the AS group. However, the constant score (SMD = 0.27; 95% CI = − 0.09, 0.63; I2 = 0%) was not significantly different between the two groups.

### Complications

**Fig. 4:**
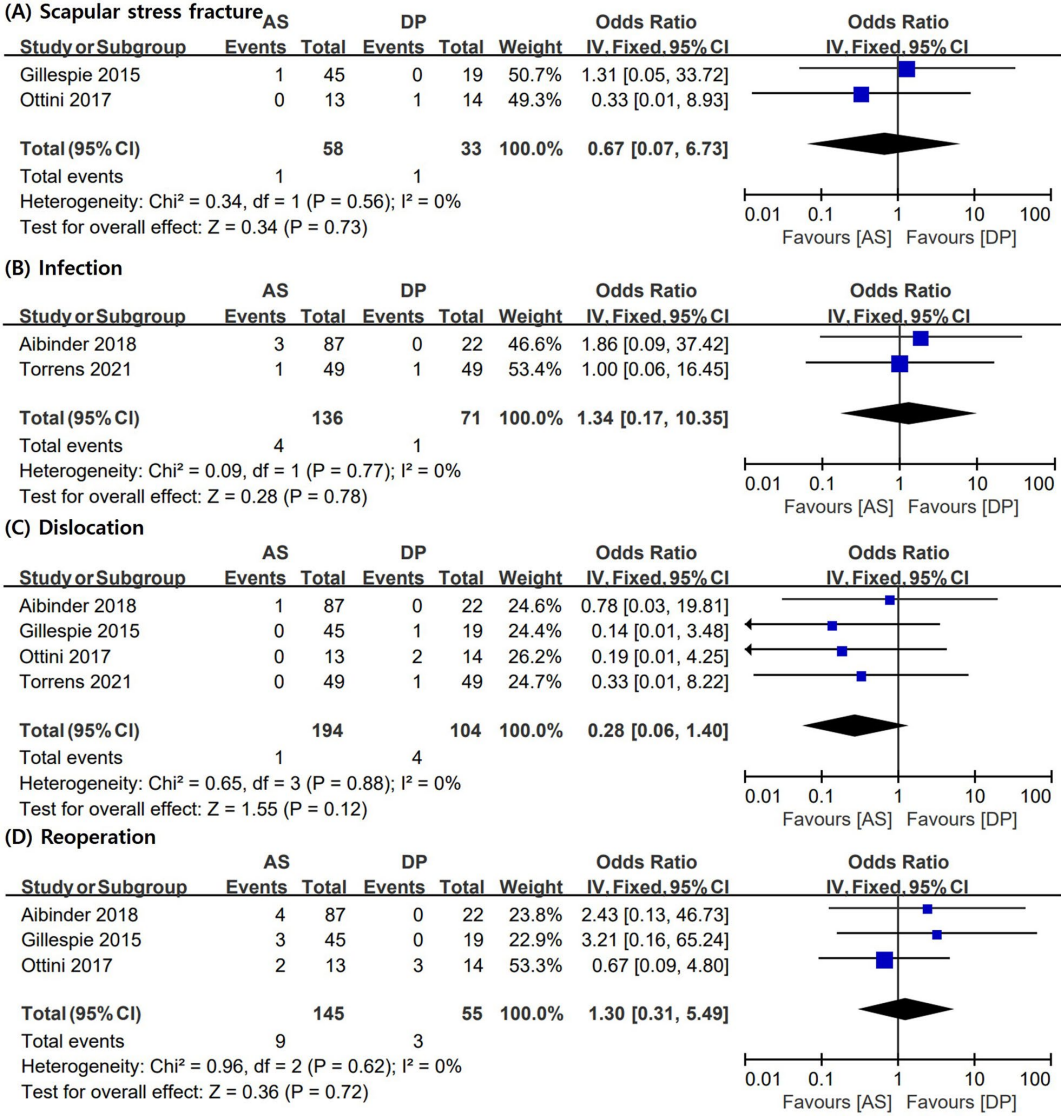
Meta-analysis results in terms of complications: **A** scapular stress fracture, **B** infection, **C** dislocation, and **D** reoperation. AS: anterosuperior approach; DP: deltopectoral approach

Several studies [3, 6, 9, 17] included in this meta-analysis reported complications, such as scapular stress fracture, infection, and dislocation. The data required for the analysis of reoperation rates are provided in three articles [3, 9, 17]. No significant difference was observed in the reoperation rate (pooled OR = 1.30; 95% CI = 0.31–5.49; I2 = 0%) between the two groups. Additionally, the incidence rates of scapular stress fracture (pooled OR = 0.67; 95% CI = 0.07–6.73; I2 = 0%), infection (pooled OR = 1.34; 95% CI = 0.17–10.35; I2 = 8%), and dislocation (pooled OR = 0.28; 95% CI = 0.06–1.40; I2 = 0%) were not significantly different between the two groups (Fig. 4).

### Publication bias

**Fig. 5:**
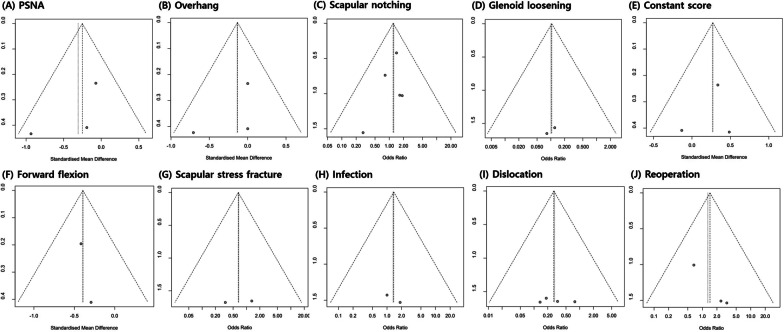
Graphic funnel plots of the included studies. PSNA: prosthesis-scapular neck angle

Funnel plot analysis was performed to assess radiological outcomes, clinical outcomes, and complications (Fig. 5). In addition, Egger’s test was performed for data reported in more than three studies. The *p*-values for all factors were > 0.05. (PSNA, *p* = 0.4487; overhang, *p* = 0.5553; scapular notching, *p* = 0.4085; constant score, *p* = 0.7634; dislocation, *p* = 0.6039; and reoperation, *p* = 0.0541).

## Discussion

RTSA is an effective and safe treatment option for glenohumeral arthropathy with massive rotator cuff tear; the procedure results in excellent pain relief and restoration of shoulder function in many patients at short-term and mid-term follow-up [18–22]. It was initially designed for the treatment of glenohumeral arthritis with a massive rotator cuff tear, but with the development of instruments and surgical instruments, it is also used for acute fracture, revision arthroplasty, and tumoral surgery, and its scope of application is gradually expanding [4, 5]. AS and DP approaches are most frequently used for RTSA. Some surgeons have argued that the DP approach is preferred over the AS approach in complex cases [6]. However, controversy remains regarding which approach is better. Several surgeons conducted a study to find an answer to this controversy, but most of the studies had only few cases and a meta-analysis has not been reported. Therefore, we performed a meta-analysis to understand which of the two approaches is better.

In this meta-analysis, the AS approach provides a lower incidence of glenoid loosening than the DP approach. In contrast, the DP approach showed better results compared with the AS approach in terms of forward flexion of the shoulder joint. No significant differences were noted in radiologic outcomes or other complication rates between the two approaches.

Achieving satisfactory radiological outcomes after surgery is of great importance during the surgical management of RTSA [23]. Therefore, many articles have evaluated radiology after RTSA [23–26]. Scapular notching is probably the most common radiologic adverse event associated with RTSA [6, 27]; in spite of efforts prevent it, it remains a cause of concern. Scapular notching has been reported to cause a statistically significant decrease in constant scores and range of motion [28]. Inferior positioning of the metaglene, which allows the glenosphere to overhang from the inferior rim of the glenoid, is one of the most effective ways to prevent it [29]. However, the AS approach is related to the superior positioning of metaglene [3, 26, 30, 31]. Levigne et al. [31] reported a significantly higher rate of scapular notching in RTSA by using the AS approach. In contrast, the surgical approach did not affect the incidence of scapular notching in our analysis. This finding is similar to the results of previous studies [8, 9].

Radiographic parameters, such as PSNA, overhang, scapular neck angle, β tilt, and peg-glenoid rim distance were used to assess the tilt and craniocaudal position. These parameters were highly correlated with the incidence of scapular notching [3]. Due to lack of studies providing accurate data for analysis, only PSNA and overhang were analyzed in this meta-analysis. The analysis of the parameters revealed no statistically significant difference between the two approaches. Consistent with the results of this meta-analysis, several previous studies [3, 6] reported no statistically significant differences in baseplate tilt.

Vanhove and Beugnies [32] reported the progression of scapular notching leading to glenosphere loosening. In the present study, no difference was observed in the incidence of scapular notching between the two groups; however, the incidence rates of glenoid implant loosening were significantly lower in the AS group compared with the DP group. The discrepancy in the frequency of scapular notching and glenoid component loosening may be because only the presence or absence of occurrence was analyzed, without reflecting the stage of scapular notching.

The clinical outcomes are closely related to patient satisfaction after surgery. Constant scores and postoperative range of motion are commonly used to evaluate the clinical outcomes. Ladermann et al. [16] noted that the mean forward flexion of the shoulder joint using the AS approach was slightly lower than that using the DP approach, although the difference was not statistically significant. Consistent with this, our meta-analysis suggested that the DP approach provides significantly better outcomes in terms of forward flexion. No significant differences were noted between the two approaches in terms of clinical outcomes when measured using the constant score in this meta-analysis. This result is in agreement with previously published studies [6, 16].

Complications, such as infection and dislocation, may lead to catastrophic results that require revision operations and need to be prevented. Several studies [33, 34] have noted that the DP approach is associated with a higher risk of dislocations because of the increased difficulty in subscapularis repair. In our meta-analysis, no significant difference was observed in the dislocation rate between the AS and DP groups. Mole et al. [8] reported a higher odds ratio for scapular stress fractures in the DP group than in the AS group. In addition, a multicenter retrospective study by Verstraete [35] noted a higher occurrence of scapular stress fracture after the deltopectoral approach. In contrast to these studies, the results of our analysis suggest no significant difference in the incidence rate of scapular stress fractures between the two groups.

### Strengths and limitations

This is the first meta-analysis to compare radiologic outcomes, clinical outcomes, and complications between the AS and DP approaches for RTSA. Additionally, all the studies included in this meta-analysis were comparative studies. The quality of the studies evaluated using the methodological index was relatively high, with a minimum score of 8.

This study has several limitations. First, a relatively small number of patients were included in the analysis. For an accurate analysis, we included only six papers in which the number of experimental and control groups were clearly described; therefore, a relatively small number of papers were included. Analysis of factors, such as peg-glenoid rim distance and beta tilt could not be performed because few studies provided appropriate data for analysis. Common study weaknesses included restricted information on the surgeons performing the surgery, perioperative care, handling of missing data, and details regarding patient selection. These factors are likely to have a major impact on clinical outcomes and complication rates.

## Conclusions

In the current meta-analysis, we compared two surgical approaches, AS and DP, for RTSA. The results of this analysis suggest that the DP approach produced significantly better outcomes than the AS approach with respect to forward flexion. In contrast, the incidence rate of glenoid implant loosening was significantly lower in the AS group than in the DP group. Both the AS and DP approaches had similar radiologic parameters (PSNA and overhang), constant score, incidence rate of scapular notching, and other complications, except glenoid implant loosening. As a result of this meta-analysis, one of the two approaches did not bring a better result. One has strength for better forward flexion and the other for a lower glenoid loosening rate. With this in mind, it is recommended to use the approach that the surgeon is most familiar with.

## Data Availability

The datasets used and/or analyzed during the current study are available from the corresponding author on reasonable request.

## Abbreviations

RTSA: Reverse total shoulder arthroplasty
AS: Anterosuperior
DP: Deltopectoral
SMD: Standardized mean difference
OR: Odds ratio
OA: Osteoarthritis
PRSIMA: Preferred reporting items for systematic reviews and meta-analysis
PSNA: Prosthesis-scapular neck angle
CIs: Confidence intervals
LOE: Level of evidence
FU: Follow-up
SNA: Scapular neck angle
PGRD: Peg-glenoid rim distance
ROM: Range of motion
VAS: Visual analog scale
ASES: American shoulder and elbow surgeon
SANE: Single assessment numeric evaluation
PSS: Penn shoulder score
NR: Not recorded
